# Isotopic Resonance Hypothesis: Experimental Verification by *Escherichia coli* Growth Measurements

**DOI:** 10.1038/srep09215

**Published:** 2015-03-18

**Authors:** Xueshu Xie, Roman A. Zubarev

**Affiliations:** 1 Division of Physiological Chemistry I, Department of Medical Biochemistry and Biophysics, Karolinska Institutet, SE-17 177 Stockholm, Sweden

## Abstract

Isotopic composition of reactants affects the rates of chemical and biochemical reactions. As a rule, enrichment of heavy stable isotopes leads to progressively slower reactions. But the recent isotopic resonance hypothesis suggests that the dependence of the reaction rate upon the enrichment degree is not monotonous. Instead, at some “resonance” isotopic compositions, the kinetics increases, while at “off-resonance” compositions the same reactions progress slower. To test the predictions of this hypothesis for the elements C, H, N and O, we designed a precise (standard error ±0.05%) experiment that measures the parameters of bacterial growth in minimal media with varying isotopic composition. A number of predicted resonance conditions were tested, with significant enhancements in kinetics discovered at these conditions. The combined statistics extremely strongly supports the validity of the isotopic resonance phenomenon (p ≪ 10^−15^). This phenomenon has numerous implications for the origin of life studies and astrobiology, and possible applications in agriculture, biotechnology, medicine, chemistry and other areas.

The four elements C, H, O and N (CHON) have fundamental importance. They are among the seven most abundant elements in our galaxy^1^. Together with helium, CHON are the five most common elements in the Solar system^2^. In Earth's crust, oxygen is the most abundant element, while C, H and N are among the dozen of most ubiquitous elements^3^. In atmosphere, CHON are among the five most abundant elements. In humans, CHON account for 96% of the body weight^4^. In bacteria, CHON compose 92% of the dry mass, and over 98% of the total living mass. CHON are the dominant elements in biopolymers, such as proteins, nucleic acids, polysaccharides and lipids. All four elements have several stable isotopes, with the lightest isotope dominating in terrestrial environment (e.g., ^2^H or deuterium atoms compose only 0.0156%, or 156 ppm, of all hydrogen atoms).

Immediately after the discovery by Urey *et al.* in 1932 of deuterium^5^, the biological effects of this heavy isotope have been intensively studied. It has been quickly found that highly enriched deuterium oxide (“heavy water”) negatively affects growth and well-being of many organisms. Large amounts of deuterium in water were found to reduce protein and nucleic acids synthesis, disturb cell division and alter cellular morphology^6^. High concentrations of deuterium were proven toxic to higher organisms^7^, although some bacteria are able to adapt to grow in almost pure heavy water^8^. There have been much fewer reports on the effect of other heavy stable isotopes in biology. In general, only high enrichments have produced statistically significant alterations. It was found possible to grow mice, sometimes for several generations, in the environment highly enriched with ^13^C^9^, ^18^O^10^, and ^15^N^11^. The heavy isotopes of C, N and O are currently considered “safe”^10^, although recently Turck *et al.* have reported that mice growth on^15^N diet exhibit systematic behavioural differences^11^. They also found that *E. Coli* grow slower in a media highly enriched with ^15^N^12^. These examples demonstrate that the effects of heavy isotopic substitution on biology are still insufficiently understood^13^. But the most poorly investigated are the effects of low enrichment levels. Conventional understanding of kinetic isotope effects postulates that, as the concentration of the heavy isotope decreases, its effect becomes progressively smaller. However, multiple violations of this postulate have been reported in literature, mostly regarding deuterium (Appendix 1). In many such reports, small deuterium concentrations gave a sizeable effect, which was in relative terms greater (and sometimes much greater) than the ratio between the deuterium and hydrogen atoms. The effects concerned not only growth of biological organisms, but also the rates of specific biochemical reactions. As an example, Lobyshev et al. have found that the Na,K-ATPase activity sharply increases at low deuterium enrichments, reaching maximum (+50% compared to normal water) at 0.04–0.05% D^14^,^15^. Lobyshev et al. understood that the effect must be collective in nature^15^, but despite the abundant experimental data, a convincing explanation for these phenomena was absent.

Recently, Zubarev et al. have formulated the Isotopic Resonance hypothesis that provides a plausible framework for these puzzling results^16^. The hypothesis predicts that at certain “resonance” abundances of the stable isotopes of C, H, N and O, the rates of chemical and biochemical reactions of certain compound classes accelerate, affecting biological growth. The proposed mechanism relates to the overall reduction of the system's complexity, understood as a total number of distinct quantum mechanical states.

The isotopic resonance conditions become obvious upon considering the normalized isotopic shift of molecular masses (NIS, the difference between the average and monoisotopic molecular masses normalized by the nominal mass, which is an integer number) plotted against the normalized monoisotopic defect (NMD, the difference between the monoisotopic and nominal masses of the molecule normalized by the nominal mass)^17^:

NMD = 1000*(Monoisotopic mass – nominal mass)/(nominal mass);

NIS = 1000*(Average isotopic mass – monoisotopic mass)/(nominal mass).

For instance, mapping masses of ca. 3,000 tryptic peptides from *E. coli* on such a 2D plot produces, besides the expected scattered “galaxy”, a gap with a line that crosses the “galaxy” (Figure 1a). The gap and the line manifest an “isotopic resonance”. The line appears due to a specific property of terrestrial isotopic compositions of CHON, while the gap is due to the fact that the peptide molecules consist of discrete number of atoms, which determines the discrete character of molecular masses, and thus of the monoisotopic defects and isotopic shifts.

Isotopic resonances, i.e. straight lines in a 2D mass plot, can be observed at many different sets of isotopic compositions. The a priori probability of such a strong feature as in Figure 1a to emerge by a random selection of isotopic abundances is ca. 1%^16^. At a resonance, the number of independent parameters describing the average mass of the molecules found on the line is reduced, which results in overall reduction of system's complexity. For example, for the molecules on the central line in Figure 1a, only six parameters are needed (four monoisotopic masses and two parameters of the line), while for the molecules outside the line, 14 parameters are required, including the masses and abundances of all stable isotopes of CHON^16^. The isotopic resonance hypothesis postulates that such a complexity reduction affects (usually accelerates) the rates of chemical and biochemical reactions. If the hypothesis is correct, then the terrestrial resonance in Figure 1a may have aided life emerging and/or taking root on our planet^16^.

The line obtained at standard terrestrial isotopic compositions is not perfect, and can be further “tuned up” to become mathematically thin. Achieving this can be done by varying the isotopic composition of any member of the CHON family; e.g., by increasing the deuterium content from the normal 0.016% to 0.03–0.06%^18^. At a perfect resonance, the rates of biochemical reactions should further increase compared to terrestrial conditions. Thus the startling effects of ultralow deuterium enrichment on molecular and biological systems^14^,^15^,^19^,^20^,^21^,^22^,^23^,^24^,^25^,^26^,^27^,^28^,^29^ can be explained by reaching the perfect resonance. On the other hand, further deviation from the perfect resonance, e.g. by depletion of deuterium in water, should slow the growth of fast-growing cells, which may explain the anticancer properties of water with depleted deuterium^30^,^31^. Interestingly, deep depletion that practically removes deuterium from consideration, decreases the system complexity compared to moderate depletion, and thus the hypothesis predicts that deep depletion should increase the reaction rates once again.

To test the isotopic resonance hypothesis, we have previously analyzed available data from published literature, and found an agreement, sometimes a remarkable one, with hypothesis' predictions^18^. Recently, we have designed a very precise (standard error ±0.05%), robotically prepared and automatically measured experiment probing the growth parameters of *E. coli* in M9 minimal media (composed only of water, glucose, ammonium chloride and inorganic salts) with varying isotopic compositions of CHON. The first study performed with the new set-up concerned the effects of low and ultralow deuterium enrichment^32^. In short, previously reported growth acceleration at ≈0.03% D has been confirmed, although in *E. coli* the effect was rather small (<1%).

Here we continue testing the isotopic resonance hypothesis on the same set-up but for other, non-terrestrial resonances. As in the deuterium study, we monitored three growth parameters that are measured independently: the lag phase duration, the maximum growth rate and the maximum density of bacteria. More comfortable growth conditions usually result in shorter lag phase, faster growth rate and higher maximum density, even though exceptions related to the last parameter have been found at >1% D^32^. Resonances are predicted for ^15^N at ≈3.5% (the standard terrestrial value is 0.37%), for ^13^C at ≈0.35% (1.1%), and for ^18^O - at 6.6% (0.2%). These resonances are expected to be of different “strengths”. One of the strongest possible resonances is predicted at simultaneous enrichment of ^13^C to 9.54%, ^15^N to 10.89% and ^18^O to 6.6%. This “super-resonance” was investigated in great detail.

## Methods

### Resonance prediction

**Figure 1:**
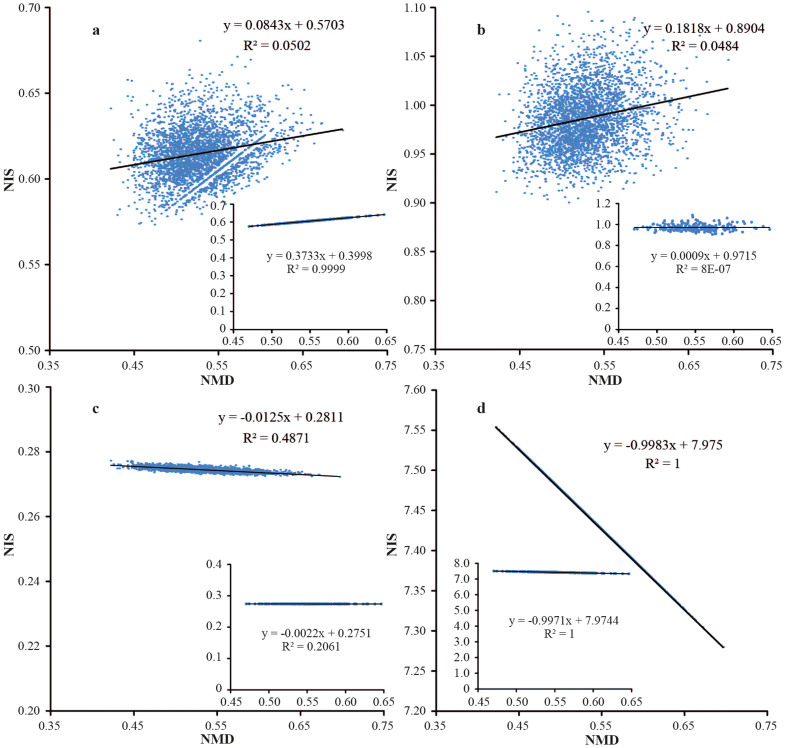
2D mass plots of 3,000 *E. coli* tryptic peptides at different isotopic ratios of CHON. Insets show only peptides with Z = 0. The axes represent: (x) normalized monoisotopic defect (NMD), and (y) normalized isotopic shift (NIS). (a) Terrestrial isotopic ratios; the gap with a central line correspond to the terrestrial isotopic resonance for Z = 0 molecules. (b) Zero-slope resonance at ≈3.5% ^15^N for Z = 0 molecules. (c) Zero-slope resonance at ^13^C ≈ 0.35% for Z = 0 molecules and a near-resonance for all molecules. (d) The “super-resonance” at ^13^C ≈ 9.5%, ^15^N ≈ 10.9% and ^18^O ≈ 6.6% for all molecules.

There is currently no rigorous theory for quantitative prediction of the position and strength of the isotopic resonances, especially for such complex systems as living organisms. However, the position and, to a certain degree, relative strength of a resonance can be predicted semi-quantitatively using a 2D mass plot as in Figure 1. The resonance occurs when a straight line is formed. The relative strength of the resonance is determined by the number of dots and the abundance of corresponding molecules on the line as well as their biological significance. When the slope of the line becomes close to zero or to a small integer value, such as 1 or −1, an additional reduction of complexity occurs, and the resonance strength is expected to increase, as the complexity decreases further. For instance, the terrestrial resonance in Figure 1a concerns molecules (not necessarily polypeptides or amino acids) following the rule: Z = 0, where Z = C − (N + H)/2^16^. Most amino acids and many polypeptides follow this rule^16^, which defines the significance of this resonance for living organisms. However, many molecules remain outside the line, which limits the strength of this resonance. Another limitation is that the linear correlation between NMD and NIS at this resonance has a non-zero and non-integer slope. Finally, at typical terrestrial isotopic abundances the line is not perfect – there is a certain spread of data, determining the line “thickness”. Thus this resonance can be improved in a number of ways, e.g., by “tuning up” the CHON isotopic abundances to a perfect resonance (ultrathin line), or by changing the line slope to zero. The zero slope can be achieved by ^15^N enrichment to ≈3.5% (Figure 1b), but the line becomes more diffuse, which should reduce the size of such an improvement. In contrast, the resonance at ^13^C ≈ 0.35% gives not only a zero slope for Z = 0, but a near-zero slope for all other molecules. The corresponding line in Figure 1c, while not perfect, is much less spread from the line compared to the “galaxy” in Figure 1a. Thus this resonance should be stronger than the ^15^N ≈ 3.5% resonance. The resonance at ^18^O ≈ 6.6% is of different nature: the average isotopic masses become proportional to the nominal (integer) masses, which totally eliminates the monoisotopic masses from the average mass equation. Such a great complexity reduction is difficult to supersede; only complete depletion of all heavy isotopes can achieve smaller complexity. The resonance at ^18^O ≈ 6.6% affects species composed of mostly hydrogen and oxygen, first of all water, the most ubiquitous and important for life molecule. Additional enrichment of other isotopes to fulfil the same resonance condition should increase the effect further. For that, ^13^C has to be be enriched from 1.1% to 10.9%, and/or ^15^N - to 9.5% (Figure 1d).

In general, extraordinary measures were taken to eliminate the possibility of human error during sample preparation, growth measurements and data processing. Every sample was compared to its own individual control grown in the neighbouring well at standard isotopic compositions. All experiments were performed in multiple replicates.

### Chemicals and materials

The bacteria were grown in M9 minimal media, with the isotopic composition varied by mixing normal ingredients with ^12^C- or ^13^C-glucose, D_2_O, H_2_^18^O or ^15^NH_4_Cl, keeping the molecular composition of the media constant. *See supplementary materials.*

***Sample preparation*** was aided by a liquid handling robot – *see supplementary materials*.

***E. coli growth measurements*** were done using an automated station - *see supplementary materials*.

***Data analysis*** was performed by a home-written computer program - *see supplementary materials.*

## Results

### Testing the ^15^N ≈ 3.5% resonance

**Figure 2:**
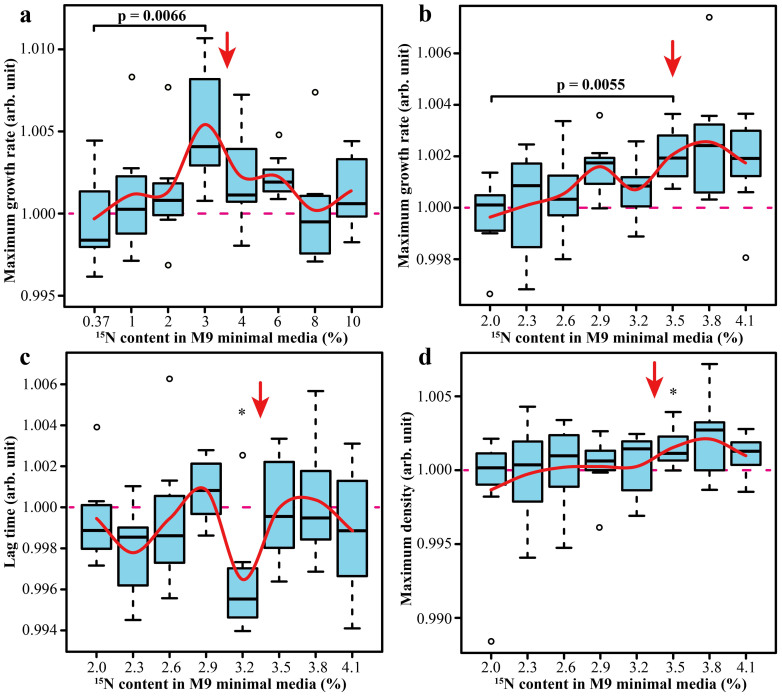
Growth parameters of *E. coli* grown in M9 minimal media with varying composition of ^15^N: (a), (b) – maximum growth rate; (c) – lag time; (d) – maximum density. In the box plots, the box encompasses 50% of the data with a central bar corresponding to a median, while the “error bars” include the remaining 50% of data except for a few data points (outliers) represented by the open circles. The red line crosses the average value in each data set.

Preliminary experiments showed that the growth rate of *E. coli* is retarded at a significant enrichment of ^15^N, which is in line with literature data^12^. For instance, at 50% enrichment, the lag time was extended by 0.53%, the growth rate decreased by 0.77%, and the maximum density was reduced by 0.94% (Figure S1). Thus the null hypothesis (based on the conventional kinetic isotope effect) expected that at ≈3.5% ^15^N enrichment, the growth of *E. coli* will be slightly suppressed: linear extrapolation of the 50% ^15^N results gave a lag time extension by 0.04%, a growth rate decrease by 0.05%, and a maximum density reduction by 0.07%. In the actual experiment, a statistically significant *increase* in the maximum growth rate was observed. Figure 2a summarizes the results of seven independent experiments where the nitrogen isotopic composition was varied from 0.37% (normal) to 10%, each experiment involving 32 sample/standard pairs for each ^15^N content point. The maximum effect was found at ≈3% ^15^N, where the growth rate increased by ≈0.5% (p = 0.007 in two-tailed Student's test). This maximum growth rate was associated with the largest spread of the data. A similar effect has been observed in the experiments with deuterium^32^. Indeed, since the initial bacterial composition was genetically and epigenetically heterogeneous, and the growth enhancement due to isotopic resonance was likely to be dependent upon the bacteria phenotype^33^, an increase in the data spread with the size of the effect was expected. In other domains of growth measurements, the lag phase showed no significant change, while the maximum growth density showed a maximum at 3%, but with a below-threshold significance (p = 0.085).

To validate the ^15^N resonance at ≈3.5%, a narrow range of ^15^N content, 2.0–4.1%, was investigated with a 0.3% step. The results (Figure 2b) confirmed the existence of a resonance around 3.5–3.8%. The size of the effect (a 0.3% increase in the maximum growth rate compared to 2.0% ^15^N, p = 0.006) was consistent with that of the broad-range experiment. The other two growth parameters were also supportive of a faster growth: the lag phase had a statistically significant minimum at 3.2%, and the maximum density was enhanced at 3.5% (Figures 2c, d). The combined p-value of these observations is <0.00005.

### Testing the ^13^C ≈ 0.35% resonance

**Figure 3:**
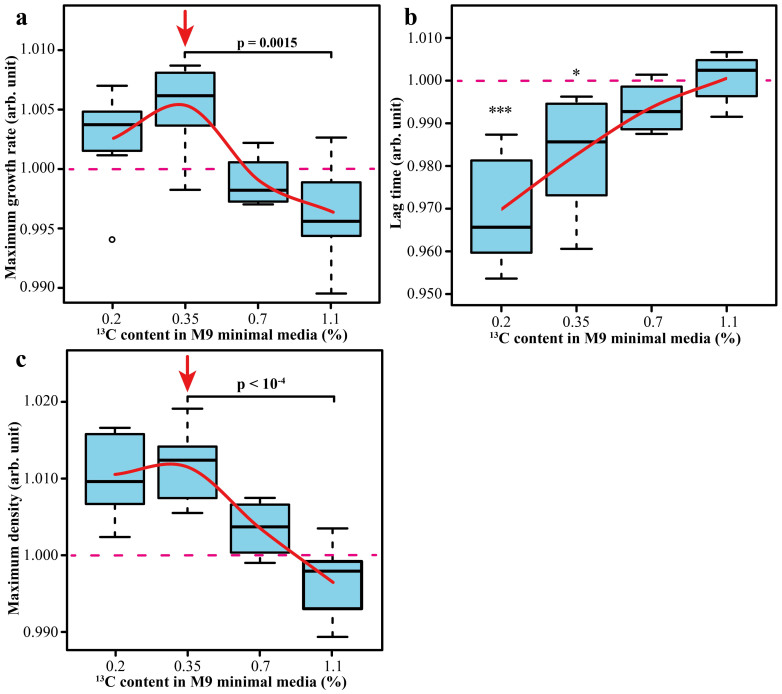
Growth parameters of *E. coli* grown in M9 minimal media with varying composition of ^13^C: (a) – maximum growth rate; (b) – lag time; (c) – maximum density.

The resonance predicted to occur at 0.35% ^13^C was tested at four different ^13^C concentrations in the range from 0.2% to 1.1% (normal terrestrial value). Figure 3 shows the results for the growth parameters. The presence of a resonance at 0.35% is supported by both maximum growth rate (+0.7%) and maximum density (+1.3%), the combined p-value being as low as 10^−6^. At the same time, the lag phase decreased strongly with the ^13^C content decrease, and reached a minimum (−3%) at 0.2% ^13^C.

**Figure 4:**
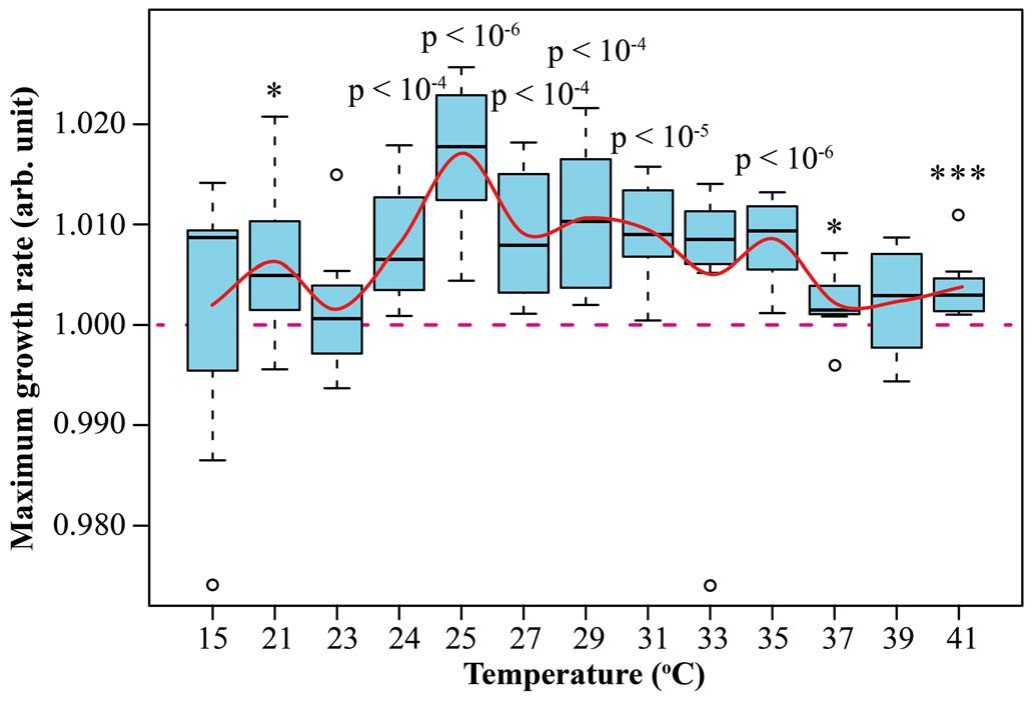
Maximum growth rate of *E. coli* grown in M9 minimal media with 0.35% of ^13^C at different temperatures.

To explore the effect of temperature on the growth rate enhancement under on-resonance condition, *E. coli* was grown in 0.35% ^13^C at a temperature ranging from 15°C to 41°C. The maximum growth rates were always higher than in isotopically normal media (the combined p-value is 10^−25^), with the largest increase, ≥1%, observed in the range between 25 and 35°C (Figure 4).

### Testing the super-resonance (^13^C = 9.54%; ^15^N = 10.89%; ^18^O = 6.6%)

**Figure 5:**
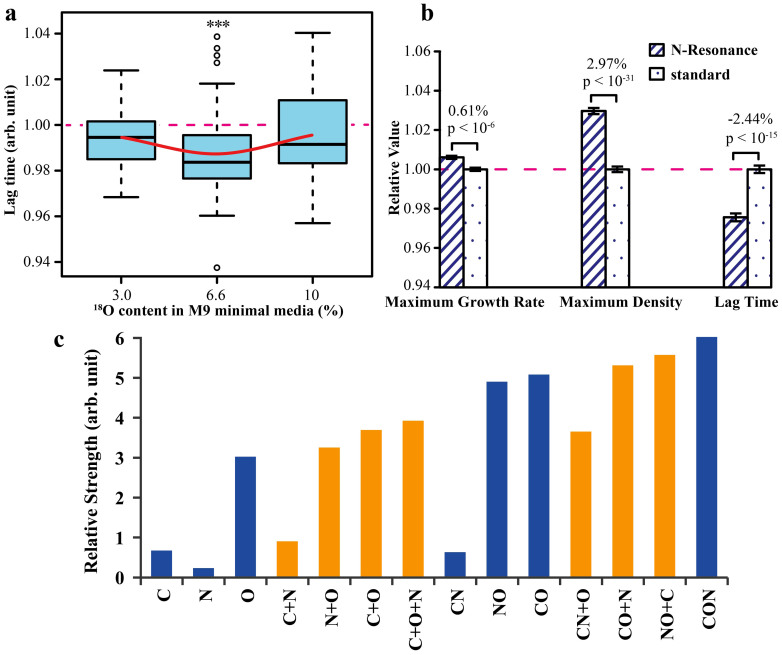
(a) Resonance in lag time at 6.6% ^18^O. (b) Resonance at the “super-resonance”: at ^13^C ≈ 9.5%, ^15^N ≈ 10.9% and ^18^O ≈ 6.6%. (c) Relative magnitudes of the effects of individual and combined isotope enrichment: blue columns – experimental results; orange columns – extrapolated data.

As a first step, we tested the resonance at ^18^O ≈ 6.6% that is valid for molecules containing H and O, i.e. water. The presence of a strong positive effect on growth is obvious in the lag phase domain (ca. −1.3%, p < 0.0002; Figure 5a). At the same time, both maximum growth rate and maximum density increase monotonously with ^18^O content at least to 10% ^18^O (Figures S2a and b). This was somewhat unexpected, given that high ^18^O enrichment is known to be detrimental for bacterial growth^10^. It is likely that, at even higher ^18^O content, both maximum growth rate and maximum density will decrease. Such experiments were not performed at this time because of the high cost of ^18^O-enriched water.

When the expected resonance at 9.5% ^13^C was tested, it was understood that enrichment of ^13^C, unlike other elements, preserves the terrestrial resonance for Z = 0. Moreover, ^13^C resonance would act mostly on molecules exceptionally rich with C and H, such as hydrocarbons, while proteins and other biopolymers might be less affected. Therefore, it was not expected that such enrichment would have a strong effect on bacterial growth. Indeed, both maximum growth rate and maximum density remained unchanged below 13% enrichment, while the lag phase was lower by 0.5% in the range of 8–13% ^13^C (Figure S3). This modest result is still consistent with the predictions of the isotopic resonance hypothesis.

At ^15^N ≈ 10.9%, there should be a resonance acting on molecules with high content of N and H, e.g., ammonia, but no increase was observed for any growth parameter (Figure S4), with all values being statistically indistinguishable from controls. This was not particularly surprising, given that the concentration of free ammonia in bacteria and growth media is low, and its role in bacterial metabolism is not particularly prominent.

Pairwise enrichments combining the above single-element resonances yielded, not surprisingly, the smallest effect for C + N (+H) and the strongest effect for C + O (+H) (Figure 5c). To compare the effects for individual and combined isotopic enrichments, the effect magnitudes of the three growth parameters were added together. Combined C + O and O + N enrichments gave larger effects than the combined effects of individual enrichments of C, O and N. But by far the largest effect was observed for the triple enrichment C + O + N (+H) (Figure 5b), which predicted to be a super-resonance for all molecules containing at least two CHON elements. At the triple enrichment, the maximum growth rate increased by 0.6%, while the maximum density - by 3%, with the lag phase shortened by 2.4%. The overall effect of triple enrichment was larger than any combination of the individual and/or pairwise enrichments.

## Discussion

Here we tested resonances predicted for C, N and O, as well as a super-resonance for these elements, while the resonance for H has been extensively tested earlier^14^,^15^,^19^,^20^,^21^,^22^,^23^,^24^,^25^,^26^,^27^,^28^,^29^,^30^,^31^. All experimental data obtained so far are either consistent with the isotopic resonance hypothesis, or strongly support it. The 15N ≈ 3.5% resonance was one of the weakest tested, but a very large volume of experimental data (two series, seven experiments in each series, with 32 sample/standard pairwise comparisons in each experiment) strongly support its validity. In contrast, the 0.35% ^13^C resonance was tested at only four different ^13^C concentrations, but it was backed up by extensive temperature series. The temperature results (Figure 4) that gave a maximum effect at 25–35°C, were rationalized through the interpretation of the isotopic resonance phenomenon suggesting that the complexity reduction leads to lower density of quantum-mechanical states, which is similar (but not equivalent) to a higher internal energy per degree of freedom, i.e. an elevated internal temperature^16^. In the range of 25–35°C, the growth rate of *E. coli* is significantly lower than the maximum rate achieved at ca. 39°C, and thus the “temperature increase” achieved via isotopic resonance has a noticeable positive effect on growth. This effect becomes progressively smaller when the temperature rises and the growth rate reaches saturation. At 39°C, the isotopic resonance effect is no longer similar to a temperature increase, but it still accelerates the growth, albeit less than at lower temperatures.

The resonance at ^18^O ≈ 6.6% was the strongest observed for any individual element, which could be explained by the relatively high degree of enrichment and the known biological effect of this isotope on microorganisms^34^. On the other hand, much stronger enrichment of ^13^C (to 9.5%) and ^15^N (to 10.9%) did not produce nearly as strong an effect as ^18^O enrichment to 6.6%. Strikingly, even though significant enrichment by any heavy isotope should lead to growth slowdown, triple enrichment led to a very strong growth enhancement. That the super-resonance conditions provide an extremely comfortable environment for bacterial growth, is perhaps the most convincing argument in favour of the isotopic resonance hypothesis.

## Conclusions and Outlook

Precise measurements of *E. coli* growth parameters at different isotopic compositions of ^13^C, ^15^N and ^18^O provided statistically significant confirmation for enhanced growth at a number of predicted resonance isotopic compositions. Taken together, these observations leave no doubts in the reality of nonlinear and resonance-like effects of the isotopic compositions on bacterial growth. Moreover, the fact that the positions of the resonances and semi-quantitative magnitude estimates are provided by the isotopic resonance hypothesis, extremely strongly suggest its validity.

**Figure 6:**
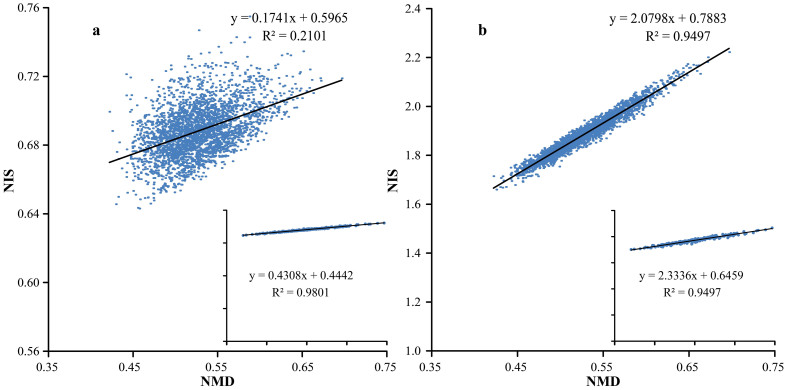
Same as in Figure 1 but for atmospheric isotopic compositions^36^ of (a) Mars, (b) Venus.

These results, combined with a wealth of literature starting from 1930s^14^,^15^,^19^,^20^,^21^,^22^,^23^,^24^,^25^,^26^,^27^,^28^,^29^,^30^,^31^, including our recent studies on the effect of enriched deuterium on bacterial growth^32^, open a venue for scientific and industrial exploration of the isotopic resonance phenomenon in a whole range of fields. In astrobiology, the impact of the isotopic resonance phenomenon on the origin of life on Earth has to be seriously considered. The atmospheric isotopic compositions of other plants of our Solar system differ from that of Earth, especially in deuterium content^35^, so that no strong resonance seem to exist on Mars or Venus (Figure 6). If this factor is linked to the probability of life, as the hypothesis suggests, searching for life on exoplanets will have an additional narrowing parameter to consider. In space exploration, growing food on the Moon or other planets may be accelerated by “tuning up” the growth environment to a convenient isotopic resonance. In biotechnology, production of biomass and biomolecules may be boosted; in chemistry, organic and perhaps even inorganic synthesis may benefit as well. It remains to be tested whether isotopic resonance can increase the rate of highly exothermic reactions, such as combustion and explosion. Food industry may also be affected, as stable isotopes are considered safe, especially at a low enrichment^10^. Last but not least, medicine applications have already been explored, albeit on a limited scale, in form of the retardation of cancer cell growth at off-resonance deuterium-depletion conditions^30^,^31^.

As a final comment, stable isotopes remain one of the few easily accessible and relatively unexplored frontiers in life sciences and technology. The validation of the isotopic resonance phenomenon will add incentive to start exploring this highly promising frontier in earnest.

## Author Contributions

R.Z. planned and supervised the experiments; provided resources; wrote the main manuscript text. X.X. performed the experiments and data processing; prepared figures and tables. Both authors reviewed the manuscript.

## Supplementary Material

Supplementary Information Supplementary Materials
