# Supplementary material for: Isotopic Resonance Hypothesis: Experimental Verification by Escherichia coli Growth Measurements
Source: Sci Rep. 2015 Mar 18;5:9215. doi: 10.1038/srep09215 (PMC4363831; doi:10.1038/srep09215)
Supplement: Supplementary Information — Supplementary Materials [file srep09215-s1.pdf]

Isotopic Resonance Hypothesis: Experimental Verification by *Escherichia coli* Growth Measurements

*Xueshu Xie[1] and Roman A. Zubarev[1]**

Supplementary Materials

1. Division of Physiological Chemistry I, Department of Medical Biochemistry and Biophysics, Karolinska Institutet, SE-17 177 Stockholm, Sweden

*Corresponding author: Roman.Zubarev@ki.se phone/fax +46 8 524 87 594

Appendix 1. Early studies of unusual biological effects of small concentrations of deuterium.

The unusual biological effects of dilute heavy water were first reported by a Yale researcher T. C. Barnes. In his experiments, mass cultures of *Spirogyra* exhibited much less abscission or cell disjunction (sign of growing at favourable conditions) and greater longevity in 0.06 % D water compared to ordinary water[19]. The positive effect of low deuterium concentration was further demonstrated in experiments confirming an increased longevity in *Spirogyra*[20]. In flatworms, longevity in the heavy water media appeared at deuterium concentrations of 0.06% and 0.07%, while at 0.13% and up to 0.47% D the effect became progressively obscure[21]. Increased cell division was observed in *Euglena* kept for forty-five days in 0.06 % heavy water[22]. Richards has repeated Barnes' tests with yeast and confirmed that a slight excess of deuterium in water is biologically significant. He observed a 26% increase in dry weight of yeast grown in 0.06% D water[23]. The strongest effect of 0.06% deuterium was observed during fast cellular proliferation[24]. The flatworm *Phagocatagracilis* left in normal water gradually shrank after a few months time to one-fifth or less of their original body size while

in those left in 0.06 %D water showed only a slight diminution[21]. Lockemann and Leunig studied the effect of heavy water with ≤0.54% D upon *Escherichia coli* and *Pseudomonasa eruginosa*. Concentrations of as low as 0.04% D were found to favour survival at adverse conditions[25].

Not all groups have confirmed the effect of diluted heavy water. Macht and Davis reported no difference between the growth in ordinary water and that with 0.06% D[26]. In fact, their data did imply a 10% faster growth, but the result was not statistically significant due to a large experimental uncertainty. Curry et al. repeated the experiments by Barnes and others but could not confirm the previously reported effects of dilute heavy water[27]. In general, early reports on the effects of diluted heavy water can be criticized for insufficient statistics and the small number of concentration points (often just two) used in the studies.

In 1970s, Lobyshev et al. have realized the deficiencies of previous efforts and studied the effects of low deuterium concentrations on biological and biochemical systems with much greater rigor. They found that the Na,K-ATPase activity increases at low deuterium concentrations, reaching maximum (+50% compared to normal water) at 0.04-0.05% D[14,15]. They also studied regeneration of hydroid polyps *Obelia geniculata* in a wide range of D_2O added to sea water. They found strong inhibition at high deuterium concentrations, as well as activation of regeneration by small (≤0.1%) deuterium concentrations[28].

In 1990s, Somlyai et al. have also shown that 0.06% deuterium in tissue culture activated the growth of L_{929} fibroblast cell lines[29]. In contrast, water with deuterium depleted below the normal levels suppressed fast-growing cells[29–31].

Chemicals and materials

Glycerol stock of *E. coli* BL 21 strain (stored at -80 ^oC) was obtained from the micorbiology lab of the department of Medical Biochemistry and Biophysics, Karolinska Institutet, Stockholm. Chemicals used to prepare M9 minimal media, including D-glucose ($C_6H_{12}O_6$, normal isotopic composition, 1.1% ^{13}C, 180.16 g/mol), disodium hydrogen phosphate ($Na_2HPO_4 \cdot 2H_2O$), monopotassium phosphate (KH_2PO_4), sodium chloride (NaCl), magnesium sulfate ($MgSO_4$), calcium chloride ($CaCl_2$), ammonia chloride (NH_4Cl, normal isotopic composition, 0.37% ^{15}N, 53.49 g/mol), were purchased from Sigma-Aldrich (Schnelldorf, Germany). Ammonia chloride containing 99.16% ^{15}N ($^{15}NH_4Cl$, 54.49 g/mol) and D-glucose containing 99% ^{13}C ($^{13}C_6H_{12}O_6$, 186.11 g/mol) were purchased from Silantes (München, Germany). D-glucose depleted with ^{13}C ($^{12}C_6H_{12}O_6$, 99.9% ^{12}C, 180.09 g/mol) was purchased from Cambridge Isotope Laboratories (Andover, MA, USA). Water ($H_2{}^{18}O$) enriched with 97% ^{18}O was purchase from Sigma-Aldrich (Schnelldorf, Germany). Select agar was purchased from Invigen (Paisley, UK). Distilled water was prepared with a Milli-Q device from Millipore (Billerica, MA, USA).

Vacuum filtration system with 0.2 µm polyethersulfone (PES) membrane for bacteria media sterilization was purchased from VWR (Stockholm, Sweden). Petri dishes (90 x 15 mm), inoculating loops and corning sterile culture tubes (16 x 125 mm) were purchased Sigma-Aldrich. Sterile plastic conical tubes (50 mL and 15 mL) for sample preparation were purchased from Sarstedt (Nümbrecht, Germany). The BioScreen C automatic fermentor was obtained from Oy Growth Curves AB Ltd (Helsinki, Finland).

Sample preparation

(1) To grow E. coli at different content of ^{15}N.

M9 minimal media preparation

5-time concentrated M9 minimal salts stock solution was prepared by dissolving 42.5 g $Na_2HPO_4 \cdot 2H_2O$, 15 g KH_2PO_4 and 2.5 g NaCl in Milli-Q water to a final volume of 1000 mL. The solution was then sterilized by autoclaving and stored at 4 ^oC for further use. To prepare M9 minimal media, the salts stock solution was diluted five times with Milli-Q water.

Nitrogen-free M9 minimal media (without nitrogen source $^{15}NH_4Cl$) were prepared by mixing the following components: 800 mL Milli-Q water, 200 mL M9 concentrated salts stock solution, 2 mL of 1 M $MgSO_4$ solution, 0.1 mL of 1 M $CaCl_2$ solution, 5 g D-glucose.

M9 minimal media with normal isotopic composition (0.37% ^{15}N, 99.63% ^{14}N) were prepared by dissolving 200.00 mg of NH_4Cl (normal isotopic composition) in 200.0 g of nitrogen-free M9 minimal media solution weighed in a sterile plastic bottle. The prepared media were then sterilized by filtering with a 200 mL vacuum filtration system through a 0.2 µm PES membrane.

M9 minimal media enriched with ^{15}N (99.16% ^{15}N, 0.84% ^{14}N) were prepared by dissolving 203.74 mg of $^{15}NH_4Cl$ (99.16% ^{15}N, 0.84% ^{14}N) in 200.0 g nitrogen-free M9 minimal media solution, followed by filtering with a 250 mL vacuum filtration system through a 0.2 µm PES membrane.

Preparation of streak agar plates

M9 minimal media agar plates with normal isotopic composition (0.37% ^{15}N, 99.63% ^{14}N) were prepared by dissolving 3 g of agar powder in 200 mL M9 minimal media (0.37% ^{15}N). The obtained mixture was sterilized by autoclaving, then cooled down to ca. 60 ^oC and finally poured into Petri dishes (ca. 15 mL agar solution per plate). The agar plates were allowed to solidify at room temperature for ca. 10 mins, sealed with parafilm and stored at 4 ^oC till further use.

E. coli streak agar plates were prepared by streaking *E. coli* from -80 ^{o}C glycerol stock onto M9 minimal media agar plate followed by ca. 40-hour incubation at 37 ^{o}C to form visible isolated colonies. Streak agar plates were stored at 4 ^{o}C for the following experiments no longer than one week. Fresh steak agar plates were prepared regularly once a week.

Preparation of E. coli sample on honeycomb well plate

From the *E. coli* agar plate, one isolated colony was picked with a sterile loop into 5 mL M9 minimal media (normal isotopic condition) and incubated at 37 ^{o}C while shaking 250 r.p.m for 5-6 hours until it reached its early exponential phase with optical density (O.D.) around 0.2, measured with Colorimeter WPA CO75 (York, UK).

Sample preparation workflow is shown in Figure S5. In each experiment, three stock solutions were used. Stock A for preparing sample S_A, and stock B for preparing sample S_B were obtained by mixing M9 minimal media with normal isotopic condition (0.37% of ^{15}N) and ^{15}N enriched M9 minimal media (99.16% of ^{15}N) at certain ratio (Table 1). M9 minimal media with normal isotopic condition (0.37% of ^{15}N) were used to prepare stock solution of standard A and standard B. The final solutions were dispensed into the honeycomb well plates using programmed robotic system (Tecan, Genesis RSP 150, Männedorf, Switzerland)

A 20 µL (36 µL for 50% of ^{15}N) aliquot of the incubated *E. coli* culture (O.D. ≈ 0.2) was diluted in 45 mL M9 minimal media of normal isotopic composition (0.37% ^{15}N) prepare diluted *E. coli* culture for robot sample preparation. First, 300 µL M9 minimal media without bacteria were introduced into each of the border wells on both plates A and B to serve as blanks (no color code in Figure S6[33]). Second, 30 µL (151 µL for 50% of ^{15}N) aliquot of stock A was dispensed into each "sample" well on plate P_A (marked with purple in Figure S6) to prepare 32 replicates of sample S_A. Third, 30 µL (151 µL for 50% of ^{15}N) aliquot of stock B was dispensed to each "sample" well on plate P_B (marked with blue in Figure S6), resulting in

32 replicates of sample B. Fourth, 30 µL (151 µL for 50% of ^{15}N) aliquot of stock solution of standards (0.37% ^{15}N) was added into each "standard" well on plate P_A and P_B (marked with yellow in Figure S6) to prepare 32 reference standards on each plate. Finally, 270 µL (149 µL for 50% of ^{15}N) of the diluted *E. coli* culture was dispensed into each well except blank wells. In total, 32 replicates pairs of "sample" and "standard" wells were prepared on each plate.

Table 1. Stock solutions and their corresponding ^{15}N compositions in the final samples. Stock solution (column one) was prepared by mixing M9 minimal media with normal isotopic composition (0.37% ^{15}N, column two) and M9 minimal media enriched with 99.16% ^{15}N (column three) at certain ratio, resulting in the final ^{15}N composition in the sample (column four).

**^{15}N composition in stock solution**	**M9 minimal media enriched with 0.37% ^{15}N (µL)**	**M9 minimal media enriched with 99.16% ^{15}N (µL)**	**^{15}N composition in the final sample (honeycomb well plate)**
0.37%	5000	0	0.37%
6.67%	4991	340	1%
16.67%	4185	827	2%
26.67%	3856	1399	3%
36.67%	3195	1856	4%
56.67%	2200	2915	6%

76.67%	1179	4000	8%
96.67%	129.0	4991	10%
16.67%	4175	825	2.0%
19.67%	4090	993	2.3%
22.67%	3883	1132	2.6%
25.67%	3776	1300	2.9%
28.67%	3614	1451	3.2%
31.67%	3491	1619	3.5%
34.67%	3311	1761	3.8%
37.67%	3152	1912	4.1%
99.16%	0	16,000	50%

(2) To grow E. coli at different content of ^{13}C.

M9 minimal media preparation

Carbon-free M9 minimal media (without D-glucose) were prepared by mixing the following components: 800 mL Milli-Q water, 200 mL M9 concentrated salts stock solution, 2 mL of 1 M $MgSO_4$ solution, 0.1 mL of 1 M $CaCl_2$ solution, 1 g NH_4Cl.

M9 minimal media with normal isotopic composition (1.1% ^{13}C, 98.9% ^{12}C) were prepared by dissolving 2000.78 mg of D-glucose (normal isotopic composition) in 400.0 g of carbon-free M9 minimal media solution weighed in a sterile plastic bottle. The prepared media were then sterilized by filtering with a 500 mL vacuum filtration system through a 0.2 µm PES membrane.

M9 minimal media depleted with ^{13}C (0.1% ^{13}C, 99.9% ^{12}C) were prepared by dissolving 500.00 mg of D-glucose (0.1% ^{13}C, 99.9% ^{12}C) in 100.0 g carbon-free M9 minimal media solution, followed by filtering with a 250 mL vacuum filtration system through a 0.2 µm PES membrane.

M9 minimal media enriched with ^{13}C (99% ^{13}C, 1% ^{12}C) were prepared by dissolving 2066.86 mg of D-glucose (99% ^{13}C, 1% ^{12}C) in 400.0 g carbon-free M9 minimal media solution, followed by filtering with a 500 mL vacuum filtration system through a 0.2 µm PES membrane.

Preparation of E. coli sample on honeycomb well plate

Sample preparation workflow is shown in Figure S5. In each experiment, three stock solutions were used. Stock A for preparing sample S_A, and stock B for preparing sample S_B were obtained by mixing M9 minimal media at normal isotopic condition (1.1% of ^{13}C) with ^{13}C depleted minimal media (0.1% ^{13}C) or ^{13}C enriched M9 minimal media (99% of ^{13}C) at certain ratio (Table 2 and Table 3). M9 minimal media at normal isotopic condition (1.1% of ^{13}C) were used to prepare stock solution of standard A and standard B. The final solutions were dispensed into the honeycomb well plates using the Tecan robot or pipettes.

To test *E. coli* growth at 0.1-1.1% ^{13}C, stock solutions were prepared according to Table 2. A 40 µL aliquot of the incubated *E. coli* culture (O.D. ≈ 0.2) was diluted in 10 mL M9 minimal media (normal) to prepare diluted *E. coli* culture. To minimize the cost of ^{13}C depleted media, pipettes were used for part of the sample preparation here. First, 400 µL M9 minimal media without bacteria were introduced into each of the border wells on both plates A and B to serve as blanks (no color code in Figure S6). Second, 360 µL aliquot of stock A was dispensed manually by pipette into each "sample" well on plate P_A (marked with purple in Figure S6) to prepare 32 replicates of sample S_A. Third, 360 µL aliquot of stock B was

dispensed to each "sample" well on plate P_B (marked with blue in Figure S6), resulting in 32 replicates of sample B. For the next step, 360 µL aliquot of stock solution of standards (1.1% ^{13}C) was added into each "standard" well on plate P_A and P_B (marked with yellow in Figure S6) to prepare 32 reference standards on each plate. Finally, 40 µL of the diluted *E. coli* culture was dispensed into each well except blank wells with robot. In total, 32 replicates pairs of "sample" and "standard" wells were prepared on each plate.

To test *E. coli* growth at 3-13% ^{13}C, stock solutions were prepared according to Table 3. A 20 µL aliquot of the incubated *E. coli* culture (O.D. ≈ 0.2) was diluted in 45 mL M9 minimal media (normal) to prepare diluted *E. coli* culture for robot sample preparation. First, 300 µL M9 minimal media without bacteria were introduced into each of the border wells on both plates A and B to serve as blanks (no color code in Figure S6). Second, 40 µL aliquot of stock A was dispensed into each "sample" well on plate P_A (marked with purple in Figure S6) to prepare 32 replicates of sample S_A. Third, 40 µL aliquot of stock B was dispensed to each "sample" well on plate P_B (marked with blue in Figure S6), resulting in 32 replicates of sample B. For the next step, 40 µL aliquot of stock solution of standards (1.1% ^{13}C) was added into each "standard" well on plate P_A and P_B (marked with yellow in Figure S6) to prepare 32 reference standards on each plate. Finally, 260 µL of the diluted *E. coli* culture was dispensed into each well except blank wells. In total, 32 replicates pairs of "sample" and "standard" wells were prepared on each plate.

Table 2. Stock solutions and their corresponding ^{13}C compositions in the final samples. Stock solution (column one) was prepared by mixing M9 minimal media depleted with ^{13}C (0.1% ^{13}C, column two) and M9 minimal media with normal isotopic composition (1.1% ^{13}C, column three) at certain ratio, resulting in the final ^{13}C composition in the sample (column four).

^{13}C composition in stock solution	M9 minimal media with 0.1% ^{13}C (μL)	M9 minimal media with 1.1% ^{13}C (μL)	^{13}C composition in the final sample (honeycomb well plate)
0.1%	15040	0	0.2%
0.2667%	12450	2490	0.35%
0.6556%	6640	8300	0.7%
1.1%	0	15040	1.1%

Table 3. Stock solutions and their corresponding ^{13}C compositions in the final samples.

Stock solution (column one) was prepared by mixing M9 minimal media with normal isotopic composition (1.1% ^{13}C, column two) and M9 minimal media enriched with ^{13}C (99% ^{13}C, column three)at certain ratio, resulting in the final ^{13}C composition in the sample (column four).

^{13}C composition in stock solution	M9 minimal media with 1.1% ^{13}C (μL)	M9 minimal media with 99% ^{13}C (μL)	^{13}C composition in the final sample (honeycomb well plate)
15.35%	5923	1009	3%
37.85%	4288	2577	6%
52.85%	3280	3678	8%
64.40%	2422	4431	9.54%

75.35%	1634	5130	11%
90.35%	604	6232	13%

(3) ***To grow E. coli at different content of*** ^{18}O

Preparation of E. coli sample on honeycomb well plate

In each experiment, four stock solutions were used. Stock A for preparing sample S_A, and stock B for preparing sample S_B were obtained by mixing M9 minimal media at normal isotopic condition with sterile ^{18}O water (97% ^{18}O) at certain ratio (Table 4). For the preparation of stock solutions of standard A and standard B, M9 minimal media were mixed with sterile Milli-Q water at the same ratio as stock A and stock B. The final solutions were dispensed into the honeycomb well plates using the Tecan robot.

A 21 µL aliquot of the incubated *E. coli* culture (O.D. ≈ 0.2) was diluted in 35 mL M9 minimal media to prepare diluted *E. coli* culture for following sample preparation. First, 300 µL M9 minimal media without bacteria were introduced with robot into each of the border wells ("edge cells") on both plates P_A and P_B (72 wells in total) to serve as blank samples (no color code in Figure S6) with robot. Second, 100 µL aliquot of stock A was dispensed into each "sample" well (marked with purple on plate P_A in Figure S6) to prepare 32 replicates of sample S_A manually by pipette. Third, 100 aliquot of stock solution of standard A was added manually by pipette into each "standard" well (marked with yellow on plate P_A in Figure S6) to prepare 32 reference standards on plate. In the same way, wells were filled on plate P_B. Finally, 200 µL of the diluted *E. coli* culture was dispensed into each well except blank wells with robot. In total, 32 replicate pairs of "sample" and "standard" wells were prepared on each plate.

Table 4. Stock solutions and their corresponding ^{18}O compositions in the final samples. Stock solution (column one) was prepared by mixing M9 minimal media with normal isotopic composition (0.2% ^{18}O, column two) and M9 minimal media enriched with ^{18}O (97% ^{18}O, column three)at certain ratio, resulting in the final ^{18}O composition in the sample (column four).

**^{18}O composition in stock solution**	**M9 minimal media (0.2% ^{18}O, μL)**	**^{18}O water (99% ^{18}O, μL)**	**^{18}O content in the final sample (honeycomb well plate)**
8.6%	3536	336	3%
19.4%	3104	768	6.6%
29.6%	2696	1176	10%

(4) To grow E. coli at ^{13}C≈9.5%, normal isotopic composition of D, ^{18}O≈6.6% and ^{15}N≈10.9%.

M9 minimal media preparation

Carbon and nitrogen free M9 minimal media (without carbon source and nitrogen source) were prepared by mixing the following components: 800 mL Milli-Q water, 200 mL M9 concentrated salts stock solution, 2 mL of 1 M $MgSO_4$ solution, and 0.1 mL of 1 M $CaCl_2$ solution.

Carbon-free M9 minimal media with 0.37% of ^{15}N were prepared by dissolving 545.00 mg NH_4Cl (0.37% ^{15}N) into 500.0 g carbon and nitrogen free M9 minimal media.

Carbon-free M9 minimal media with 99.16% of ^{15}N were prepared by dissolving 555.19 mg NH_4Cl (99.16% of ^{15}N) into 500.0 g carbon and nitrogen free M9 minimal media.

M9 minimal media with 1.1% of ^{13}C and 0.37% of ^{15}N were prepared by dissolving 1362.50 mg D-glucose (normal isotopic composition) into 250.0 g carbon-free M9 minimal media with 0.37% ^{15}N. The prepared media were then sterilized by filtering with a 250 mL vacuum filtration system through a 0.2 µm PES membrane.

M9 minimal media with 26.4% of ^{13}C and 31.9% of ^{15}N were prepared by dissolving 1010.12 mg D-glucose (1.1% of ^{13}C) and 364.02 mg of D-glucose (99% of ^{13}C) into 250 g carbon-free M9 minimal media with 31.93% of ^{15}N which were prepared by mixing 170.1 g carbon-free M9 minimal media with 0.37% ^{15}N and 79.9 g carbon-free M9 minimal media with 99.16% ^{15}N, followed by filtering with a 250 mL vacuum filtration system through a 0.2 µm PES membrane.

Preparation of E. coli sample on honeycomb well plate

Stock A: M9 minimal media with 26.4% of ^{13}C, 31.9% of ^{15}N and 19.4% of ^{18}O were prepared by mixing 860 µL sterile ^{18}O water (97% of ^{18}O) with 3475.8 µL M9 minimal media with 26.4% of ^{13}C and 31.9% of ^{15}N (prepared above).

Stock solution for Standards: M9 minimal media with normal isotopic composition (1.1% of ^{13}C, 0.37% of ^{15}N, 0.2% of ^{18}O) were prepared by mixing 860 µL sterile Milli-Q water with 3475.8 µL M9 minimal media with 1.1% of ^{13}C and 0.37% of ^{15}N (prepared above).

A 10 µL aliquot of the incubated *E. coli* culture (O.D. ≈ 0.2) was diluted into 15 mL M9 minimal media (1.1% of ^{13}C and 0.37% of ^{15}N) to prepare diluted *E. coli* culture for following sample preparation. First, 300 µL M9 minimal media without bacteria were introduced into each of the border wells ("edge cells") on P_A serve as blank samples (no color code in Figure S6) with Robot. Second, 100 µL aliquot of stock A was dispensed into each "sample" well (marked with purple on plate P_A in Figure S6) to prepare 32 replicates of sample S_A manually by pipette. Third, 100 aliquot of stock solution of standards was added manually by pipette into each "standard" well (marked with yellow on plate P_A in Figure S6) to prepare 32 reference standards on plate. Finally, 200 µL of the diluted *E. coli* culture was dispensed into each well except blank wells with the robot. In total, 32 replicate pairs of "sample" and "standard" wells were prepared on the plate.

E. coli growth measurements

After sample preparation on the honeycomb well plate, *E. coli* concentration in each well was continuously monitored by measuring turbidity (with wide band filter 420–580 nm) using Bioscreen C instrument with continuous shaking at 39 ^{o}C. Turbidity was sampled every six minutes and was monitored for ca. 22 hours to obtain a raw growth curve.

Data analysis

Data analysis[33] was performed with Excel software as described in reference 33.

Using Microsoft Excel, the logarithm of turbidity was plotted against time. The slope for every 8-h interval was calculated, and the maximum value was determined. The extrapolation of the line with maximum slope to the background level of turbidity gave the lag time. The maximum turbidity for each replicate was taken as the maximum density. The obtained three values for each growth curve were treated in the same way as below.

For each "sample" A, the obtained value was normalized by that of the "standard" B. To minimize the influence of nonstatistical outliers that could arise due to gross errors in sample preparation and handling (e.g. differences in the geometry of the honeycomb wells, position-dependent sensitivity of the BioScreen C detector, etc.), the 32 replicates were divided into 4 groups according to their positions on the honeycomb well plate (group 1: columns 1 and 2; …, group 4: columns 7 and 8). In each group, the median of the eight values was calculated and then the four medians were averaged to obtain the value for a given plate and its standard deviation.

Altogether, seven independent 32-replicate experiments were performed for each ^{15}N content point for the experiment to test *E. coli* growth at 0.37-10% ^{15}N; seven independent 32-replicate experiments were performed for each ^{15}N content point for the experiment to test *E. coli* growth at 2.0-4.1% ^{15}N; seven independent 32-replicate experiments were performed for each ^{13}C content point for the experiment to test *E. coli* growth at 0.1-1.1% ^{13}C; five independent 32-replicate experiments were performed for each ^{13}C content point for the experiment to test *E. coli* growth at 3-13% ^{13}C and three independent experiments were performed to test *E. coli* growth at the super resonance condition (^{13}C = 9.54%; ^{15}N = 10.89%; ^{18}O = 6.6%). The final result was obtained when the average of 7x4 = 28 (take experiment of testing *E. coli* growth at 0.37-10% ^{15}N as example) median values, and the corresponding standard error, were calculated. The p-values for non-terrestrial compositions were calculated using two-tailed, paired Student's t-test against the terrestrial composition (standard).

Supplementary Figures

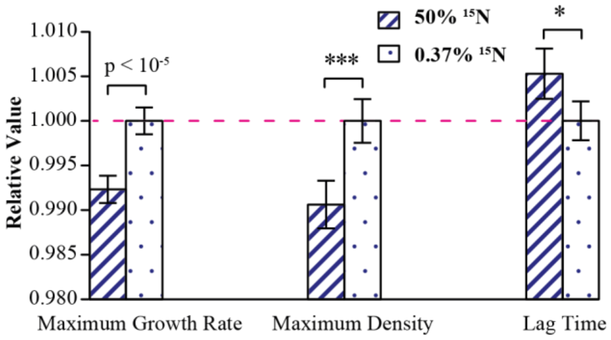

Figure S1. Growth parameters of *E. coli* at 50% of ^{15}N in M9 minimal media, compared to normal isotopic conditions.

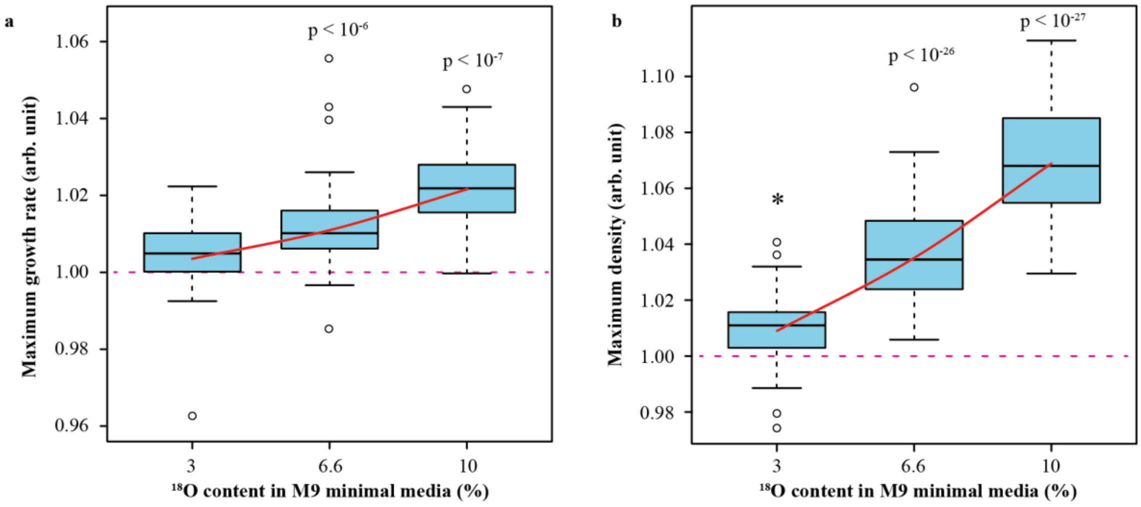

Figure S2. Maximum growth rate (a) and maximum density (b) of *E. coli* growth with ^{18}O enriched in the water of M9 minimal media.

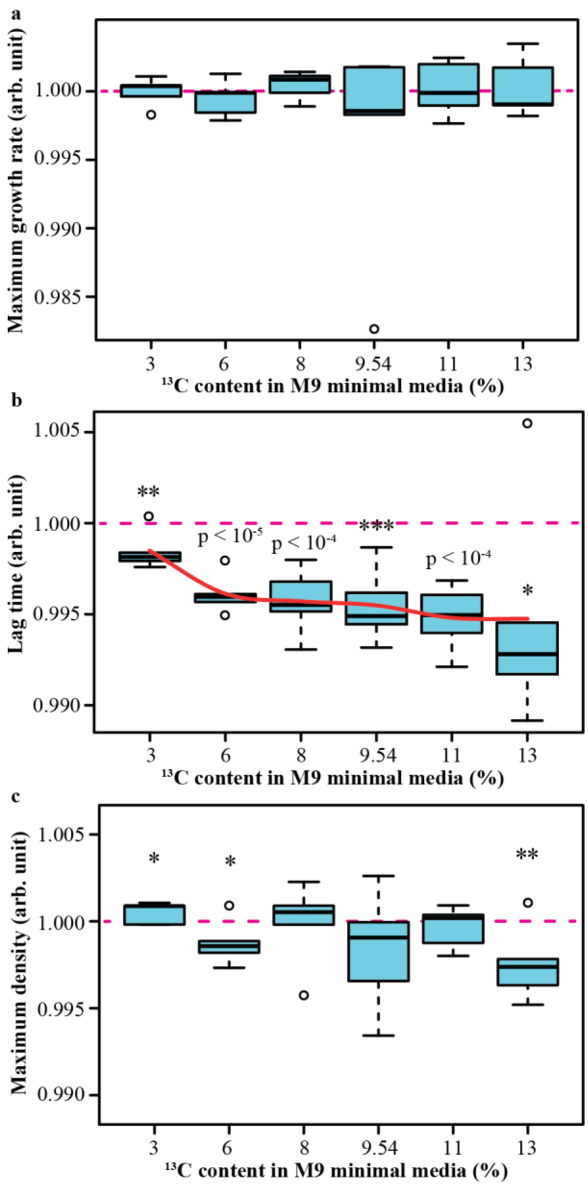

Figure S3. Growth parameters of *E. coli* growth with ^{13}C enriched in the glucose of M9 minimal media. (a) Maximum growth rate, (b) lag time, (c) maximum density.

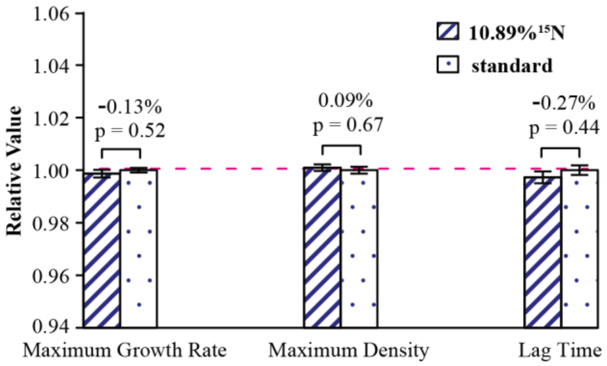

Figure S4. Growth parameters of *E. coli* growth with ^{15}N enriched in the salt of M9 minimal media.

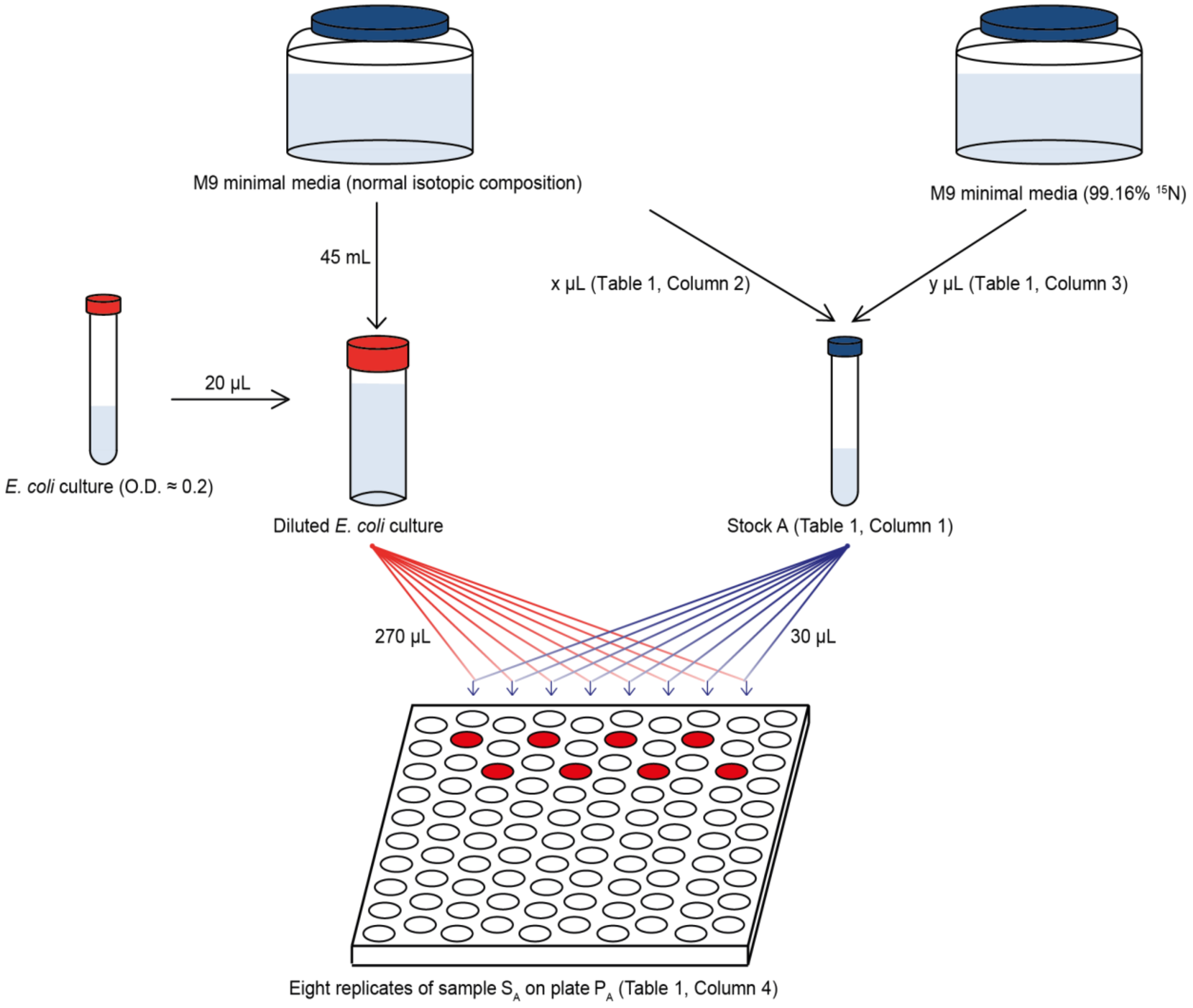

Figure S5. Workflow of sample preparation.

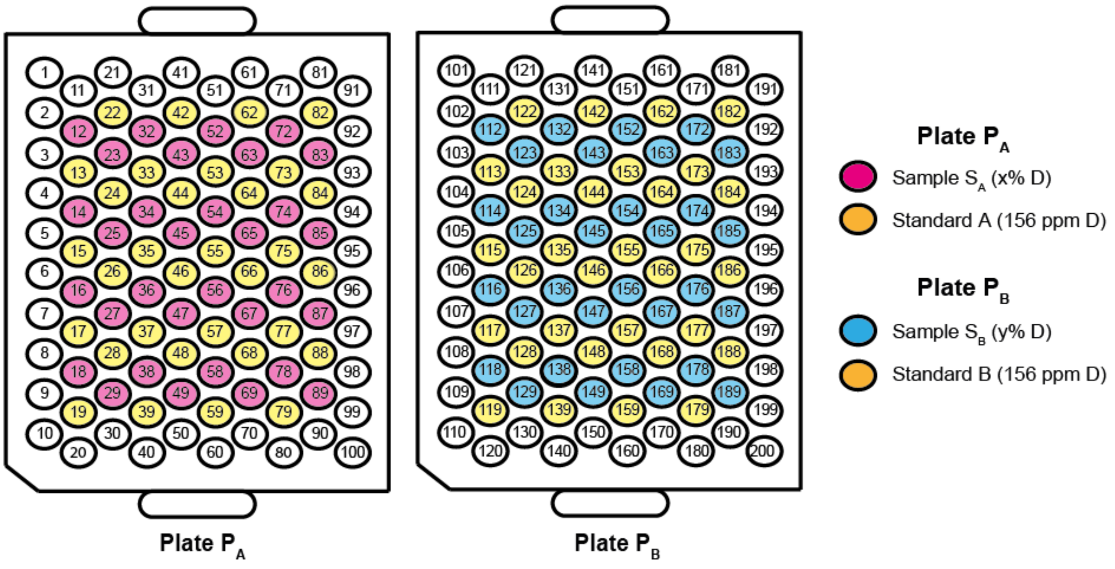

Figure S6. Sample configuration on the honeycomb well plates, adapted from reference 32.
